# Animal–Visitor Interactions: Where Do We Go from Here?

**DOI:** 10.3390/ani16193035

**Published:** 2026-09-26

**Authors:** Eduardo J. Fernandez, Claudia C. Martinez, James Edward Brereton, Andrew Moss

**Affiliations:** 1 School of Animal and Veterinary Sciences, Adelaide University, Adelaide, SA 5005, Australia; claudia.martinez@adelaide.edu.au; 2 Department of Psychological Science, University of Texas Rio Grande Valley, Edinburg, TX 78539, USA; 3 Higher Education, University Centre Sparsholt, Westley Lane, Sparsholt, Winchester SO21 2NF, UK; james.brereton@sparsholt.ac.uk; 4 Chester Zoo, Chester CH2 1LE, UK; a.moss@chesterzoo.org

**Keywords:** animal–staff interactions, animal–visitor interactions, anthropogenic effects, aquariums, human–animal interactions, visitor–animal interactions, visitor effects, visitor experiences, zoos

## Abstract

Animal–visitor interactions (AVIs) involve the study of both the impacts of zoo visitors on animals (visitor effects) and those of animals on zoo visitors (visitor experiences). In the last century, particularly in the last few years, AVIs have become an increasingly studied topic within zoos. However, most of this research has focused on correlational studies of the visitor effect on the behavioral welfare of mammal species. In this paper, we discuss additional considerations for future AVI research that could assist in how we study AVIs, including research incorporating: (1) animal–staff interactions, (2) non-mammalian species, and (3) improved methods and metrics used. With all three, past and potential future research are considered, including what might help in incorporating such factors. Thus, the goal is to provide the reader with a better understanding of additional AVI research factors worth consideration, as well as offering a path forward for how to advance and expand our AVI research efforts.

## Simple Summary

Animal–visitor interactions (AVIs) involve the impacts of zoo animals and visitors on each other. AVIs have become one of the most studied research topics in zoos, in part because of the ease with which non-intrusive observational studies of AVIs can be conducted at a zoo. However, although current observation-based AVI research is relatively easy to conduct, it lends itself to certain limitations, particularly as a result of the lack of diversity in the species studied and the means by which AVI research is often conducted. As a result, this paper looks to suggest new directions for future AVI research, which could include staff considerations, greater species diversity, and new methods and metrics used within AVI research. The inclusion of such variables for study are considered, particularly as they relate to our understanding of AVIs in zoos.

## 1. Introduction

Human–animal interactions (HAIs) in zoos and aquariums often involve interactions between exhibited animals and visitors, or what has been called animal–visitor interactions (AVIs; [1,2,3,4,5]). AVIs are often grouped by the presumed impact of interest [6], including the impact of the visitor on the animal (i.e., visitor effects; [7,8]), and the impact of the animal on the visitor (i.e., visitor experiences; [9,10]). Over the last several decades, we have seen an increase in the study of AVIs in zoos, particularly over the last few years [11]. This increase has included greater diversification of the species studied, which historically were more likely to focus on zoo primate species [12,13,14], as well as greater interest in the types of visitor experiences involved, including conservation education [15,16,17]. In addition, although most AVI research in the past focused on negative visitor effects, there has been an increasing trend in studying visitor effects that are linked to positive welfare [8,11,18]. The results of these changing trends have led to greater diversification in how AVIs are examined, including the subjects involved and methods of study [14,19,20].

Although clear advances have been made in the study of both HAIs and AVIs in zoos, a number of common trends in their study limit the applicability of any results. For instance, others have pointed out that much of the current published literature relies almost exclusively on correlational, rather than experimental, methods [6,8,11,14]. As such, possible incorrect attributions can occur, such as causal assumptions of visitor effects on the welfare of animals (for instance, see [21]). In only a few cases have we seen AVI variables directly manipulated, such as the access, activity, density, or direct view of an exhibit by visitors [22,23,24,25]. This has also led to investigators examining factors outside the animal–visitor dichotomy found to influence AVI results, including temperature and weather [26,27,28]. Similar limitations exist for the typical AVI animal subjects studied, which, although diversified from their historical reliance on zoo primates, still focus largely on mammalian species [11,14]. For continued advancement of AVI research to occur, there needs to be critical evaluation of these trends, including novel suggestions for how to progress.

In this paper, we examine new ways of examining AVI research, particularly as related to the human–animal interaction variables of interest and how they are measured/manipulated. This is conducted through three suggestions for future research expansion that include: (1) animal–staff interactions; (2) non-mammalian species; and (3) improved methods and metrics used. The first two variables deal specifically with who is studied (i.e., staff and non-mammalian species), while the third variable focuses on what and how AVI-related variables are measured. For all three possibilities, we consider both past research that has successfully incorporated such variables, as well as considerations for greater implementation. The goal is to foster increased diversification in the ways in which AVI research is examined, thereby increasing our ability to both validate and generalize our findings.

## 2. Animal–Staff Interactions

Although animal–staff interactions are technically not part of AVIs, they share many similarities in terms of potential welfare effects on the exhibited animals [29,30]. However, unlike AVIs, only a handful of studies have looked at animal–staff interactions in zoos, one of the most comprehensive of those studies being Kathy Carlstead’s [31] examination of keeper–animal relationships (KARs) with four species of animals (black rhinoceros, *Diceros bicornis*; cheetah, *Acinonyx jubatus;* maned wolf, *Chrysocyon brachyurus;* and great hornbill, *Buceros bicornis*) across 46 zoos. The study included keeper questionnaires, a keeper calling test (response time to a keeper calling the animal), and various behavioral and physiological measures of animal welfare. Although many of the findings were species-dependent, several consistencies emerged. For three of the species, fear of people and affinity to keepers were positively correlated, in that, as fear went up or down, so did affinity for keepers (presumably related to the importance of the relationship the animal has with staff as opposed to strangers) and vice versa. For instance, most black rhinos in the study had both a high affinity to keepers and fear of people, suggesting that the only human interactions that interested the rhinos were with familiar individuals. Possibly most relevant, however, was that interactions with keepers tended to be better when occurring with some form of barrier, and that keeper job satisfaction tended to go up as fear of people in animals went down. Barriers themselves can be considered a form of protected contact, which itself is often associated with the use of reward-based training methods [32,33]. Likewise, handling procedures themselves are often associated with potential stress in other settings, such as with production animals [34,35]. Similarly, the use of coercive methods (i.e., contrived negative reinforcement and positive punishment) to attempt to manage or otherwise change behavior is commonly associated with undesired behavior, such as avoidance or aggression [36,37,38], as well as limitations on an animal’s ability to exhibit choice and control [39]. Therefore, it is critical that we better understand how keeper attempts to change animal behavior itself might influence welfare.

Several other studies have attempted to empirically examine animal–staff interactions. For instance, Ward and Melfi [40] looked at response times to animals being called by keepers based on whether they were fully trained, partially trained, or untrained using positive reinforcement. They found that animals that were fully trained using positive reinforcement had the shortest response latencies, which they associated with reductions in fear. Ward and Melfi [41] would later go on to examine how the formation of keeper–animal dyads themselves, and their individual variation influenced how animals responded. Finally, both Greco et al. [42] and Carlstead et al. [43] examined animal–staff interactions in both African (*Loxodonta africana*) and Asian (*Elephas maximus*) elephants. Although the former study mostly focused on demographic factors associated with the management and care of zoo elephants, including their interactions with keepers, the latter Carlstead study showed that, as the strength of keeper–elephant bonds went up, so did keeper satisfaction and elephant welfare. As such, the overall benefit of improving animal–staff interactions suggests a win–win scenario for the staff and the animals.

As mentioned above, only a few animal–staff interaction studies exist. One of the possible reasons for this limited study number is, of the handful of published studies thus far, most have relied on multi-facility collaborations using complex measures. However, this certainly need not be the case, particularly when reliant on small sample size (small-N; see Section 4.1) studies, as is commonly the case in zoos [44,45,46,47]. Likewise, some of the simpler measures used in standard AVI studies could apply to examinations of animal–staff interactions, including how staff ratings of their relationships with different animals correlate to their time spent with the animal (similar to visitor length of stay measures) and the behavioral responses of the animals. More detailed discussions about possible metrics are considered in Section 4 below, but our simple point here is that there is considerable need for increased animal–staff interaction studies, and therefore those considering some type of visitor-focused AVI study might consider examining staff interactions as well.

## 3. Non-Mammalian Species

Historically, the focus of AVI research has been on mammals, particularly primate species [13]. Although more recently, we have seen greater movement away from primate species, the majority of publications still focus on mammalian species [11,14]. For instance, Williams et al. [14] found that, of the 105 papers studying 252 non-primate species, more than half of these non-primate species (56%) still focused on mammalian taxa, with only 16% of all species studied being non-bird, non-mammal species. Similarly, Lin et al. [11] found that, of the 157 total AVI papers reviewed, mammals made up over 77% of all species studied, and birds made up another 13%, leaving less than 10% of all other taxa studied in AVI publications. Not surprisingly, this matches the general trend within all animal welfare studies conducted in zoos and aquariums, where mammals have been found to be the focus of ~75% of all animal welfare-focused research [48].

The importance of understanding the impacts of visitors on non-mammalian species cannot be understated. For instance, touch tanks remain a fairly common visitor experience, particularly in aquariums, yet only three published studies, to date, have examined the welfare impact of these interactions [49,50,51]. One reason for this might be that touch tanks generally house invertebrates, which have only recently gained greater welfare interest [52,53]. Nonetheless, visitors have considerable impacts across all exhibited zoo taxa, and yet the research has focused almost exclusively on understanding these visitor impacts on mammals.

As one taxonomic example, reptiles have had only six total AVI papers published, all focused on visitor effects [28,54,55,56,57,58]. All six papers have been published within the last decade, with most papers focused on multiple species. Additionally, all papers found mixed results in terms of potential negative to positive welfare impacts from visitors. This was further complicated by how positive or negative welfare was understood in terms of the different reptile species. For instance, Hanson et al. [58] found that snakes showed greater activity and entropy scores (enclosure variability) when stressed by visitors, rather than as a sign of positive welfare. In addition, the absence of visitors altogether may have produced greater stress than their regular occurrence, which suggests caution should be taken in how we change visitor traffic, rather than simply the size or intensity of the crowds themselves.

The critical point is that, for AVI research to gain greater generalizability, it needs to be more expansive in the species studied, including for visitor experience research focused on visitor attitudes and perceptions of non-mammalian species [59]. Likewise, the greater the diversity of species in AVI research, the better the validity of our findings, particularly as they relate to our understanding of different species needs from a welfare perspective. This is also important as we expand our AVI research efforts into understanding possible positive or enriching visitor effects [1,7,8], which are more frequently being measured in recent AVI research [11]. Greater taxonomic diversity may also require researchers to consider whether commonly used AVI measures have equivalent welfare relevance across taxa. For example, behavioral measures such as increased activity or enclosure-use variability may have different welfare implications depending on the species and context [58]. Future AVI research should therefore consider not only increasing the representation of non-mammalian species, but also identifying and validating species-appropriate behavioral, physiological, and space-use measures for interpreting visitor effects. This is particularly important for taxa that may perceive visitors in a substantially different way than mammals, limiting the ability to generalize the majority of visitor effect research to such species. Overall, there is a great need to study both the visitor effect and experience in relation to less typically studied zoo species, and future AVI research could help build better bridges between species-focused welfare needs and human–animal interactions.

## 4. Improved Methods and Metrics Used

The use of metrics to study the visitor effect on the welfare of animals, as well as overall methodological design, has, to date, largely been limited to behavioral measures used in correlational designs. The use of physiological measures has been less frequent, with most studies involving cortisol measured in mammals [14,60,61,62,63]. In addition, most behavioral measures have been limited to activity budgets/ethograms [11,14]. However, other behavior-based measures, including behavioral diversity [64,65] and enclosure use variability [66,67], have assisted welfare assessments in identifying a wider use of behavioral repertoires and exhibit space usage, respectively. This is additionally aided by the use of computer applications for measuring behavioral welfare, such as the ZooMonitor program and its add-on, the Enclosure MonitoR Use (EMU) app [68,69].

Below, we consider three specific areas in relation to method and metric inclusion: (1) methods used, including single-case designs; (2) visitor variables; and (3) environmental and other variables. In the case of the first, the use of single-case designs as methodological consideration is critical for taking experimental advantage of small-N designs typically used in zoos. In the case of the last, inclusion of other non-visitor variables, such as temperature and weather, as noted in the Introduction, can help eliminate potential confounds otherwise attributed to visitor effects [26,27]. In all cases, the use of such methods and metrics should facilitate our ability to properly manipulate and understand variables associated with AVIs, particularly when other factors may play important roles in causing the observed effects.

### 4.1. Small-N Designs, Longitudinal Measurement, and Mixed-Effects Methods

Despite calls for greater methodological rigor, much AVI research remains analytically conservative, and often relies on statistical approaches that do not fully capitalize on the detailed longitudinal and repeated-measures datasets often generated in zoo and aquarium settings. This is somewhat surprising, given that zoo research is uniquely positioned to benefit from analytical approaches that explicitly account for individual variation and repeated observations, such as mixed-effects modeling [47,70]. Mixed-effects models and other modern analytical techniques are now widely established across many related disciplines, yet their adoption within parts of the AVI literature remains inconsistent. Greater usage of these approaches would not only improve the validity of statistical inference, but also help the field move towards a more nuanced understanding of how individual animals respond to specific visitor characteristics and environmental contexts.

Longitudinal approaches could further strengthen AVI research by establishing individual baseline patterns and examining whether responses to visitors remain stable or change over time. Previous longitudinal AVI research has demonstrated the value of monitoring animals across changes in visitor conditions, including zoo closures and reopenings, for examining potential habituation and re-habituation to visitors [71]. Such designs could help determine whether responses to visitors change with repeated exposure and whether these responses vary across environmental, social, or seasonal contexts.

In addition, and for pragmatic reasons pertaining to the typically small number of individuals exhibited, zoos are often limited to small-N designs. Although this is often presented as a limitation due to the minimization of external validity, it likewise presents an argument for greater experimental control, and thus increased internal validity [72,73]. Specifically, the use of single-case designs, where a few subjects are run repeatedly through controlled conditions, often including reversals of those conditions, offers the ability to directly test potential causal variables [44,45,46,74,75]. Thus, single-case designs provide strong experimental control, which is ideal for better understanding factors that may influence AVIs. For instance, and as previously noted in the Introduction, several studies have taken advantage of using single-case repeated-measures designs to study the effects of directly manipulated visitor factors, including visitor activity, presence, and visibility, on the behaviors of exhibited zoo animals [22,23,24,25]. In addition, the use of mixed-effects methods, noted above, allows researchers to incorporate inferential statistics without worry of violating independent sample assumptions imposed by single-case designs [47].

The end result is that greater use of single-case designs, longitudinal measurement, and mixed-effects models would allow AVI researchers to incorporate experimental methods into their studies, and thus help minimize accidental attributions of cause imposed by otherwise observation-only techniques [76]. In addition, single-case designs do not impose large subject sample size requirements that are often prohibitive for most zoo welfare science [44].

### 4.2. Visitor Variables

In addition to the considerably lower proportion of AVI studies (<15%) that measure visitor experiences [11], a notable limitation of much AVI research relates to the way visitor-related variables are measured. Although animal responses are often quantified in considerable detail through behavioral, physiological, and welfare indicators, visitors themselves are frequently represented using relatively crude proxy measures such as visitor density, exhibit attendance, or overall zoo visitation rates [9]. This creates a notable asymmetry in AVI research; namely, one side of the interaction is characterized with the needed complexity, while the other is reduced to simple measures of presence, absence or abundance. For example, a crowd of thirty adults may present a very different sensory and behavioral stimulus to an animal than a similarly sized group of school children, yet both may be recorded identically within many study designs.

Factors such as visitor behavior, detectability (for the exhibited animal), movement patterns, noise levels, group composition, dwell time, engagement with exhibits, and the nature of interactions occurring at the enclosure may be more important determinants of animal responses than visitor numbers alone. However, only a few studies to date have attempted to directly manipulate visitor behavior to study visitor effects on animal welfare, including variables such as active versus passive audiences and perceived height [22,77,78]. To further strengthen this, animal and human measures should be measured concurrently, where both visitor effects and experiences could simultaneously be observed. Consequently, attributing observed behavioral or welfare outcomes to generic “visitor effects” without adequately characterizing what those effects are may limit the validity of findings crucial to animal welfare. Further expansion of visitor-related factors could categorize such variables in terms of quantity (e.g., count; density), composition (e.g., sex; age), behavior (e.g., activity type; activity level), and so on, the point being that we should expect that visitor-related effects and experiences are impacted and moderated by the type of visitor involved.

An additional consideration is that commonly measured visitor characteristics, such as visitor number, may not fully represent the nature or intensity of an animal’s actual exposure to visitors [25,79]. For example, visitor number alone may not adequately capture differences in visitor-generated noise or other characteristics of visitor activity [28,79]. Visitor conditions may also be associated with changes in animal behavior, enclosure use, and distance from visitor viewing areas [28,80]. Future AVI research could therefore examine whether animals alter their horizontal or vertical space use under different visitor conditions, including whether they change their distance from viewing areas, use elevated areas, retreat indoors, position themselves behind visual barriers, or otherwise alter their visibility or proximity to visitors. Consequently, future studies could measure visitor characteristics concurrently with animal location and enclosure use to determine not simply whether visitors are present, but the extent to which individual animals are exposed to, approach, or avoid them. This may be particularly important when comparing animals housed in exhibits that differ in the availability of retreat areas or opportunities for animals to exercise choice and control over their exposure to visitors [25,39]. Such approaches could help distinguish the presence of potentially aversive visitor stimuli from an animal’s behavioral response to, and ability to control, that exposure.

Visitor conditions may also influence interactions among animals, rather than affecting individuals in isolation. For socially housed species, future research could examine whether changes in visitor activity, noise, or other visitor characteristics correspond with changes in inter-individual proximity, affiliative behavior, aggression, or selection of particular social partners. Social relationships have been shown to moderate physiological responses to challenging conditions in other contexts; for example, wild chimpanzees exhibited lower urinary glucocorticoid levels when interacting with or in the presence of bond partners compared with other individuals [81]. Future AVI research could therefore examine whether animals increase proximity to or affiliation with preferred social partners during potentially challenging visitor conditions and whether these relationships buffer behavioral or physiological responses to visitors. Examining these group-level responses could expand AVI research beyond the traditional visitor–individual animal relationship to consider how visitor conditions influence broader social dynamics.

### 4.3. Environmental and Other Variables

The impact of environmental variables on zoo animal behavior has been well documented, including factors such as noise; temperature; humidity; weather, and enclosure variables, such as exhibit complexity and location [82,83,84,85,86]. However, as previously noted, a few studies have looked more directly at how AVIs are affected by such environmental variables such as weather, temperature, and humidity, finding that the visitor effect might be overestimated when accounting for such external factors [26,27,28]. In another recent example, researchers found that, although active behaviors and vocalizations increased for Asian small-clawed otters (*Aonyx cinereus*) in the presence of more visitors, they were also weakly positively correlated with time, increasing closer to the end of the day [87].

Given the established importance of enclosure complexity and design for zoo animal behavior and welfare [84], future AVI research could examine whether physical characteristics of exhibits and visitor viewing areas similarly moderate animal responses to visitors. Previous AVI research has experimentally manipulated visitor viewing proximity or access [24,25], providing a foundation for further investigation of how spatial characteristics shape AVIs. For example, an equivalent number of visitors may represent substantially different exposure conditions depending on the distance between visitors and animals, exhibit size, indoor versus outdoor location, viewing barriers, vertical complexity, and the availability of multiple areas or retreat spaces. Future research could also examine whether indoor and outdoor environments differ in fundamental exhibit characteristics (e.g., overall size; lighting; structural differences), visitor proximity, crowd size, and an animal’s opportunities for avoidance, particularly when areas differ substantially in size, visibility, or access to retreat spaces. Comparing animal responses across different areas within the same exhibit, or across conditions in which animals have differing opportunities to move away from visitor viewing areas, could help researchers determine how exhibit design interacts with visitor variables. Such research may be particularly valuable for understanding whether access to greater environmental choice and control moderates behavioral or welfare responses associated with visitors [25,39].

Of importance here is that AVI researchers should spend more time measuring environmental and other external variables, so as to avoid neglecting their possible influence and ability to moderate observed visitor effects and experiences. This is particularly relevant given that most AVI research tends to be correlational, which makes the ability to confound observations with visitor variables that much more likely. In addition, for taxa such as reptiles and amphibians, which are particularly sensitive to environmental variables such as temperature and humidity, it becomes much more critical that we understand how potential visitor effects interact with such factors. As AVI research continues to grow and advance as a relevant zoo research topic, it will be critical for AVI researchers to also adapt their observations to include non-visitor variables, along with those that are specifically AVI-focused.

## 5. Discussion

AVI research has steadily increased over the last few decades, with an exponential increase in the last few years [11,63]. This has included increases in non-primate mammal studies, as well as the inclusion of positive welfare indicators as a result of the visitor effect [8,14]. This increasing trend has made AVI research one of the most studied topics in zoological settings, helping shape exhibited animal welfare assessment and improvement, as well as guiding visitor education and entertainment [10,15].

Nonetheless, although AVI research has grown and diversified in terms of the species and variables studied, it has still largely been limited to studies involving indirect visitor interactions, and primarily in terms of the effect of the visitor on the behavioral activity of animals [4,88]. As discussed throughout this review, AVI research could greatly expand its understanding of human–animal interactions in zoos through greater inclusion of animal–staff interaction studies, which should likewise improve the husbandry and care of zoo animals and assist in assessing and improving staff satisfaction. Additionally, AVI examinations that included non-mammalian species should help with the generalizability of AVI results, as well as help identify species-specific welfare needs in zoos. Finally, greater use of diverse methodology, such as single-case designs and mixed-effects methods; greater diversity in how visitors are measured; and inclusion of additional variables, such as environmental factors, should broaden the ability to run AVI studies. This can and should include greater measurement of a diversity of visitor-related variables, including the concurrent measurement of visitor experiences (how animals impact the visitor) along with visitor effects. As a result, acknowledging and incorporating these AVI research factors could help advance its impact as an applied scientific endeavor.

One additional commentary that was only briefly previously mentioned is the inclusion of technology to assess and improve zoo animal welfare. Others have argued for such greater technological use throughout zoos [89], and there have additionally been examples of researchers using technology such as computer projections or touchscreens to understand and improve AVIs [90,91]. Technology itself could serve as a vehicle to improve all the factors mentioned as avenues for improved AVI research, allowing for greater facilitation of future AVI studies and their related practices.

## 6. Conclusions

AVI research continues to grow as a central research topic in zoos. Along with this expansion comes a greater need for the types of interactions studied, including those involving staff; increased examinations involving a diversity of taxa, particularly non-mammalian species; and increased inclusion of the methods and metrics used to study AVIs, including experimental methodology and the incorporation of non-visitor variables. Such approaches could help identify practical strategies for modifying visitor behavior while determining whether these changes correspond with improvements in animal welfare. Through greater incorporation of these factors, we are more likely to understand and improve human–animal interactions that occur in zoos, assist in our assessment and improvement of animal welfare, and facilitate how we provide for visitor education and staff satisfaction. Ultimately, our attempts to advance and expand how we study AVIs should provide a means for improving the exhibition of zoo animals as a whole.

## Data Availability

No data are associated with this publication.
